# A ferrocene redox-active triazolium macrocycle that binds and senses chloride

**DOI:** 10.3762/bjoc.8.25

**Published:** 2012-02-13

**Authors:** Nicholas G White, Paul D Beer

**Affiliations:** 1 Chemistry Research Laboratory, Mansfield Road, Oxford, OX1 3TA, United Kingdom

**Keywords:** anion binding, C–H···anion interactions, electrochemistry, ferrocene, triazolium

## Abstract

A ferrocene bis(triazole) macrocycle was synthesised in good yield by the Eglinton coupling of an acyclic bis(alkyne) precursor and characterised in the solid state by X-ray crystallography. Alkylation gives the corresponding triazolium macrocycle, which binds chloride and benzoate strongly in CD_3_CN solution through favourable charge-assisted C–H···anion interactions, as evidenced by ^1^H NMR titration experiments. Preliminary electrochemical studies reveal that the redox-active macrocycle is capable of sensing chloride in CH_3_CN solution.

## Introduction

The copper(I)-catalysed cycloaddition of alkynes and azides (CuAAC) [1–2] to give the 1,2,3-triazole group is increasingly being exploited in the synthesis of a vast array of materials since its discovery [3]. Several seminal studies have demonstrated the ability of acyclic and macrocyclic bis- and poly(triazole) containing systems to bind anions in organic solvents through triazole C–H···anion interactions [4–7]. More recently, we [8], and others [9–10], have shown that alkylating the triazole group to give the triazolium group increases the strength of anion binding significantly by further polarising the C–H bond of the heterocycle.

With one notable recent exception of an acyclic ferrocene-appended aryl triazole receptor, which selectively senses phosphate species electrochemically in dichloromethane [11], to the best of our knowledge, redox-active triazole and triazolium receptors are unprecedented. Herein we describe the synthesis of a novel ferrocene bis-triazolium macrocyclic receptor and investigate its anion binding and electrochemical-sensing properties.

## Results and Discussion

### Synthesis

**Scheme 1:**
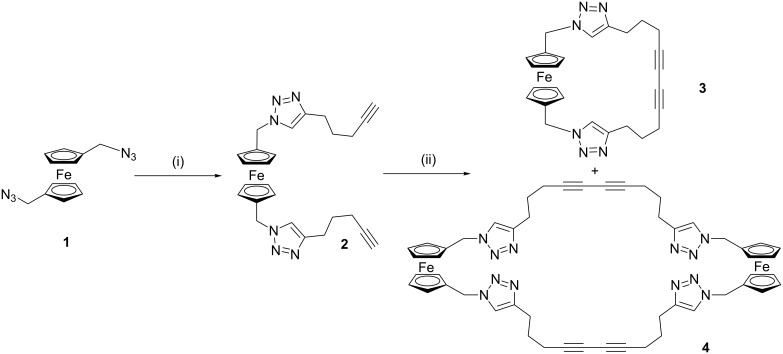
Synthesis of bis(triazole) macrocycle **3** and tetra(triazole) macrocycle **4**. Conditions and reagents: (i) 1,6-Heptadiyne, [Cu^I^(CH_3_CN)_4_](PF_6_), tris[(benzyltriazol-4-yl)methyl]amine (TBTA), diisopropylamine (DIPEA), CH_2_Cl_2_, 50%; (ii) Cu^II^(OAc)_2_·H_2_O, CH_3_CN, reflux, 54% of **3**, 12% of **4**.

The CuAAC reaction of ferrocene bis(azide) **1** [12], with a large excess of 1,6-heptadiyne afforded **2** in 50% yield. An intramolecular Eglinton cyclisation reaction was used to prepare the ferrocene bis(triazole) macrocycle **3**, in surprisingly good yield (54%) after purification by column chromatography, as well as a small amount (12%) of the corresponding larger tetra(triazole) macrocycle, **4** (Scheme 1).

**Scheme 2:**
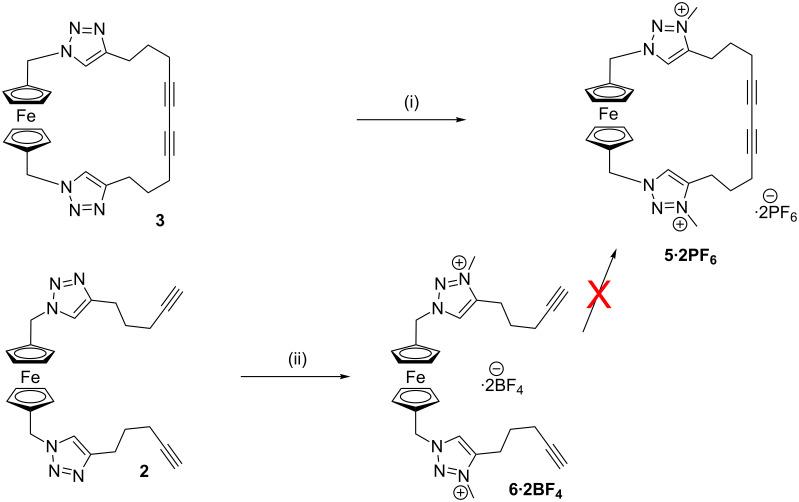
Synthesis of bis(triazolium) macrocycle, **5**. Conditions and reagents: (i) (a) (Me_3_O)(BF_4_), CH_2_Cl_2_, (b) chromatography with 17:2:1 CH_3_CN:H_2_O: sat. KNO_3(aq)_, then NH_4_PF_6(aq)_, 12%; (ii) (Me_3_O)(BF_4_), CH_2_Cl_2_, 77%.

The reaction of macrocycle **3**, with an excess of trimethyloxonium tetrafluoroborate gave a crude product, which was difficult to purify. Chromatographic purification was required, but this was complicated by the doubly positively charged macrocycle. Nevertheless, preparative thin-layer chromatography in 17:2:1 acetonitrile:water:saturated KNO_3(aq)_ solution, followed by removal of the organic solvents under reduced pressure and precipitation of the product as its bis(hexafluorophosphate) salt from the remaining aqueous solution (using NH_4_PF_6_), gave the desired bis(triazolium) macrocycle **5**, in moderate yield (Scheme 2). Alternatively, **2** could be readily alkylated to give bis(triazolium)-bis(alkyne), **6**, in good yield; however, attempts to cyclise under analogous conditions to those used to produce **3** were unsuccessful, resulting in decomposition of the ferrocene motif.

**Figure 1:**
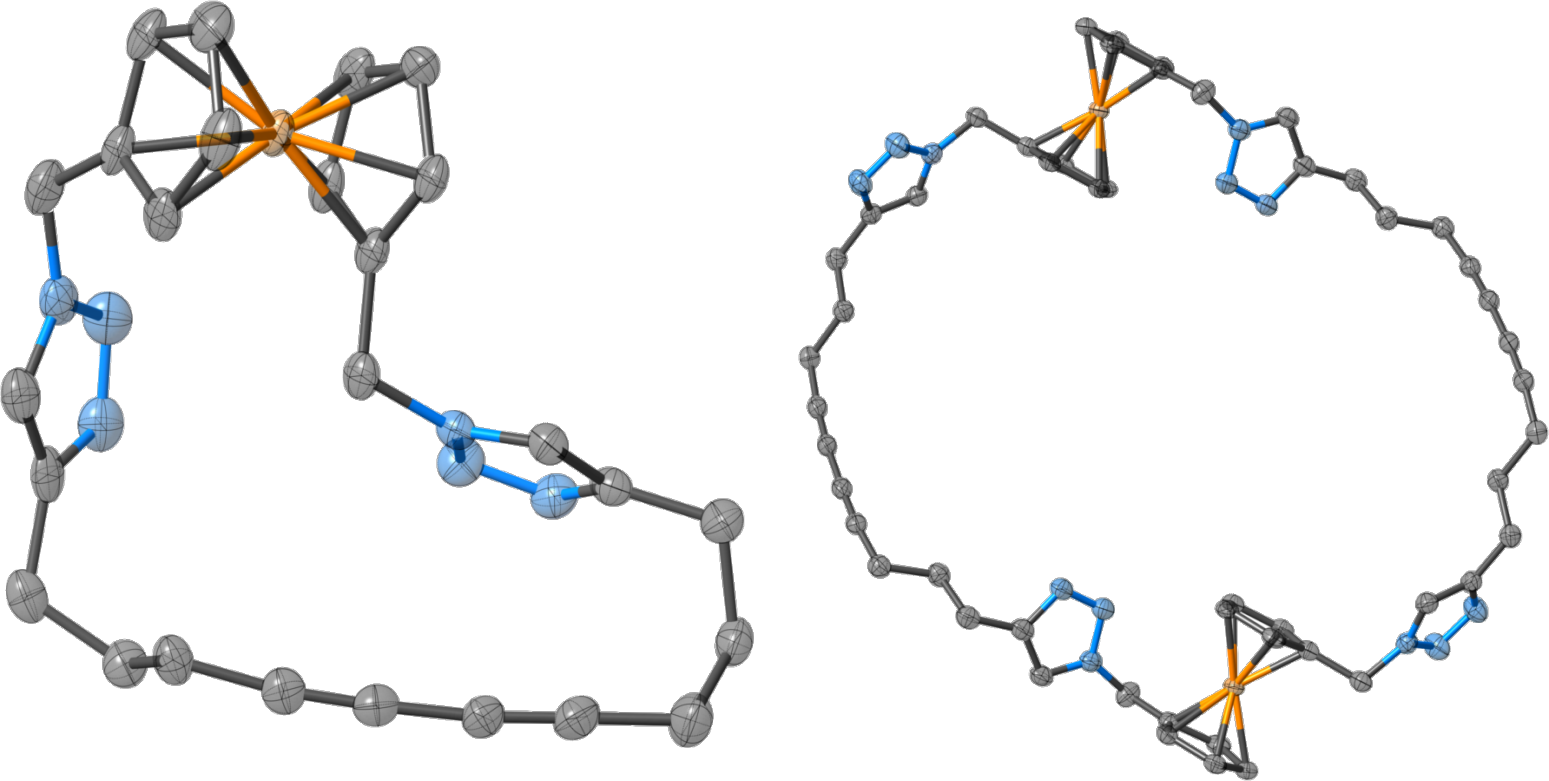
Solid-state structure of **3** (left) and **4** (right). Hydrogen atoms omitted for clarity, ellipsoids are shown at 50% probability; only one of the two independent molecules in the asymmetric unit of **3** is shown. Colour scheme: grey = carbon, blue = nitrogen, orange = iron.

All new compounds were characterised by ^1^H and ^13^C NMR spectroscopy and high-resolution ESI mass spectrometry, as well as by ^19^F and ^31^P NMR spectroscopy where appropriate. Single crystals of **3** were grown by the vapour diffusion of diethyl ether into an acetone solution of the macrocycle, and of macrocycle **4** by vapour diffusion of pentane into a chloroform/methanol solution. The solid-state structures were determined by synchrotron X-ray crystallography (Figure 1). The structure of **3** shows that the diyne unit appears to enforce a reasonably rigid geometry on the rest of the macrocycle, due to its sterically demanding nature, which may help to constrain the anion binding cleft and lead to selective anion binding. In contrast, the larger size of **4** gives a much more open structure, with a diameter of approximately 17 Å (Figure 1). Despite this open structure, no solvent cocrystallises with the macrocycle, with the structure being tightly packed due to a series of intermolecular hydrogen bonds between triazole C–H donors and triazole N-acceptors on adjacent molecules.

### Anion-binding investigations

**Figure 2:**
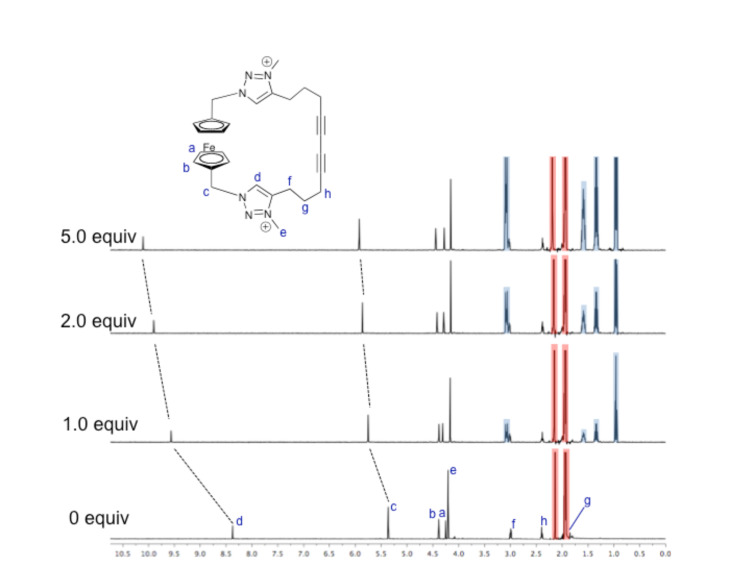
^1^H NMR spectra of **5**·**2PF_6_** after the addition of 0, 1.0, 2.0 and 5.0 equiv of TBA·Cl (500 MHz, 293 K, CD_3_CN). Red-shaded peaks correspond to solvent signals (H_2_O and CH_3_CN); blue-shaded peaks correspond to signals from TBA cations.

The anion-binding properties of **5**·**2PF_6_** were investigated in CD_3_CN solution by ^1^H NMR titration experiments. Aliquots of anions as their *N*-tetrabutylammonium (TBA) salts were added to **5**·**2PF_6_** and the chemical shift of the (chemically equivalent) triazolium protons was monitored. As shown in Figure 2, the addition of chloride to the macrocyclic receptor resulted in large downfield shifts of the triazolium signal, d, concomitant with relatively smaller perturbations of proton c, which suggests that halide binding occurs in proximity to the ferrocene group.

**Figure 3:**
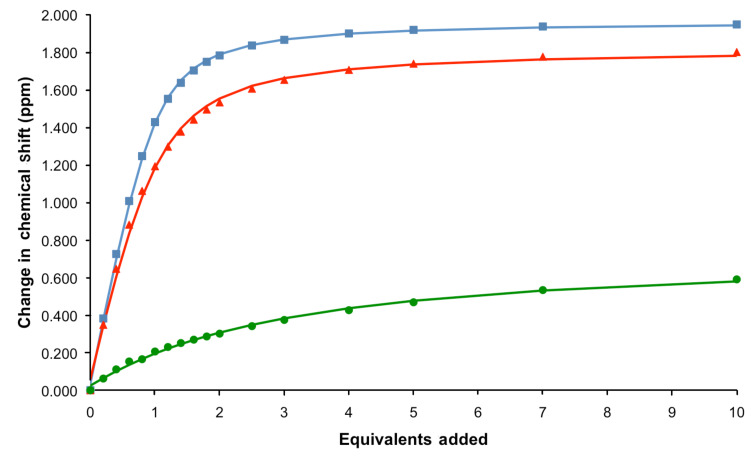
Titration data (solid points) and fitted binding isotherms (curves) monitoring the triazolium proton, d, for titration of benzoate, chloride and iodide into a solution of **5**·**2PF_6_** (500 MHz, 293 K, CD_3_CN).

WINEQNMR2 [13] analysis of the titration data (Figure 3) shows that the cyclic receptor, **5**·**2PF_6_**, binds both chloride and benzoate strongly with 1:1 stoichiometries in CD_3_CN (association constants are shown in Table 1), with the more basic carboxylate anion forming the strongest complex. The larger halide, iodide, is only weakly bound. The addition of small amounts (<1 equiv) of dihydrogen phosphate caused precipitation and, hence, no association constant could be calculated.

**Table 1:** Anion association constants for **5**·**2PF_6_** calculated by using WINEQNMR2 [13] based on ^1^H NMR titration data (500 MHz, 293 K, CD_3_CN).

Anion^a^	*K*_a_ (M^−1^)^b^

Benzoate	4.6(2) × 10^3^
Chloride	2.5(2) × 10^3^
Iodide	2.0(2) × 10^2^
Dihydrogen phosphate	Precipitation

^a^All anions added as TBA salts. ^b^Estimated standard errors given in parentheses.

### Electrochemical Investigations

**Figure 4:**
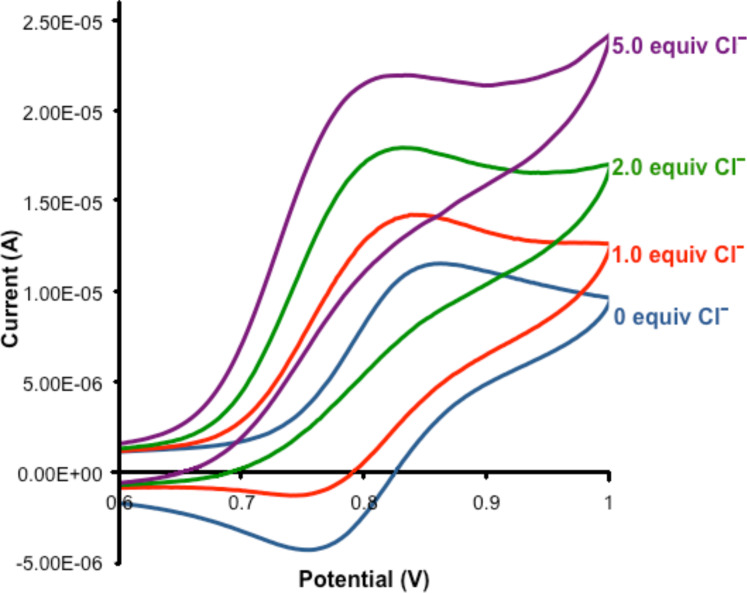
CV of **5**·**2PF_6_** upon the addition of TBA·Cl (electrolyte: 0.1 M TBA·PF_6_/CH_3_CN, [**5·2PF_6_**] = 0.50 mM, 293 K. Potential vs. Ag/AgCl reference).

The ability of **5**·**2PF_6_** to sense anions electrochemically was investigated by cyclic voltammetry in 0.1 M TBA·PF_6_ in CH_3_CN. The macrocycle displays a quasi-reversible oxidation of the ferrocene/ferrocenium couple, which is shifted to a more positive potential by 0.38 V relative to ferrocene by virtue of the positively charged triazolium groups of the receptor. Addition of one equiv of TBA·Cl caused a cathodic shift of the oxidation potential of 25 mV and a loss of reversibility, which may be indicative of an EC mechanism [14] (Figure 4). Further addition of TBA·Cl led to increasing cathodic perturbations of the E_pa_ oxidation wave of up to 40 mV after five equiv. As noted with other ferrocene based anion receptors [15–17], chloride-anion binding stabilises the ferrocenium oxidation state, which results in the cathodic perturbation observed. Analogous electrochemical experiments with benzoate caused an immediate disappearance of the redox signal, which indicates precipitation of an insoluble ferrocenium complex upon oxidation.

## Conclusion

A new ferrocene bis(triazole) macrocycle was readily synthesised by intramolecular Eglinton coupling of an acyclic 1,1’-bis(triazolylalkyne) ferrocene precursor. Alkylation of this macrocycle gave the dicationic bis(triazolium) macrocycle, which was demonstrated by ^1^H NMR titration experiments to bind chloride and benzoate in CD_3_CN, solely through charge-assisted C–H···anion hydrogen-bonding interactions. The redox-active macrocycle was also shown to sense chloride electrochemically via a cathodic shift of the E_pa_ wave of the ferrocene/ferrocenium redox couple.

## Experimental

### General Remarks

Bis(azide) **1** [12], and TBTA [18] were prepared as described in the literature. Dry dichloromethane and acetonitrile were purged with nitrogen and passed through a MBraun MSP-800 column. Water was deionised and microfiltered in a Milli-Q^®^ Millipore machine. Tetrabutylammonium salts were stored in a vacuum desiccator under reduced pressure. All other compounds were bought commercially and used as received. Routine NMR spectra were recorded on a Varian Mercury 300 spectrometer with ^1^H NMR operating at 300 MHz, ^13^C at 75.5 MHz. Spectra for anion-binding titrations were recorded on a Varian Unity Plus 500 spectrometer with ^1^H operating at 500 MHz. Mass spectra were recorded on a Bruker microTOF spectrometer.

### Bis(alkyne) **2**

Bis(azide) **1** (0.148 g, 0.500 mmol) and 1,6-heptadiyne (0.69 mL, 0.55 g, 6.0 mmol) were dissolved in dichloromethane (50 mL). DIPEA (0.17 mL, 0.13 g, 1.0 mmol), TBTA (0.053 g, 0.10 mmol) and [Cu^I^(CH_3_CN)_4_](PF_6_) (0.037 g, 0.10 mmol) were added, and the yellow solution was stirred at room temperature under a nitrogen atmosphere for three days. It was then taken to dryness under reduced pressure and puriﬁed by column chromatography (silica, 2% methanol in dichloromethane) to give **2** as a yellow solid. Yield: 0.120 g (50%). ^1^H NMR (CDCl_3_) δ 7.24 (s, 2H, trz-*H*), 5.19 (s, 4H, Fc-C*H*_2_-trz), 4.23 (t, ^3^*J* = 1.6 Hz, 4H, Fc-*H*), 4.19 (t, ^3^*J* = 1.6 Hz, 4H, Fc-*H*), 2.80 (t, ^3^*J* = 7.6 Hz, 4H, trz-C*H*_2_-CH_2_), 2.22 (dt, ^3^*J* = 7.0 Hz, ^4^*J* = 2.6 Hz, 4H, C*H*_2_-C≡CH), 1.95 (t, ^4^*J* = 2.6 Hz, 2H, C≡C*H*), 1.87 (m, 4H, trz-CH_2_-C*H*_2_); ^13^C NMR (CDCl_3_) δ 147.3, 120.6, 83.9, 82.7, 69.9, 69.6, 69.0, 49.5, 28.1, 24.6, 18.0; HRMS–ESI (*m*/*z*): [M + Na]^+^ calcd for C_26_H_28_N_6_Fe·Na, 503.1617; found, 503.1614.

### Bis(triazole) macrocycle **3**

The bis(alkyne) **2** (0.120 g, 0.250 mmol) and copper(II) acetate monohydrate (0.125 g, 0.625 mmol) were heated under reﬂux in acetonitrile (150 mL) under a nitrogen atmosphere overnight. The green solution was cooled to room temperature and then taken to dryness under reduced pressure. It was taken up in water (20 mL) and extracted with dichloromethane (4 × 20 mL). The combined organic fractions were dried (magnesium sulfate), taken to dryness under reduced pressure and then puriﬁed by column chromatography (silica, gradient: 3% methanol in dichloromethane) to give **3** as an orange powder. Yield: 0.065 g (54%). ^1^H NMR (CDCl_3_) δ 7.44 (s, 2H, trz-*H*), 5.06 (s, 4H, Fc-C*H*_2_-trz), 4.15 (t, ^3^*J* = 1.9 Hz, 4H, Fc-*H*), 4.10 (t, ^3^*J* = 1.9 Hz, 4H, Fc-*H*), 2.91 (t, ^3^*J* = 6.6 Hz, 4H, trz-C*H*_2_-CH_2_), 2.31 (t, ^3^*J* = 6.6 Hz, 4H, C*H*_2_-C≡C), 1.92–2.01 (m, 4H, trz-CH_2_-C*H*_2_); ^13^C NMR (CDCl_3_) δ 146.9, 121.9, 110.9, 84.0, 69.3, 69.1, 66.6, 49.1, 26.6, 24.2, 18.4; HRMS–ESI (*m*/*z*): [M + Na]^+^ calcd for C_26_H_26_N_6_Fe·Na, 501.1461; found, 501.1467.

### Tetra(triazole) macrocycle **4**

The same procedure as for the synthesis of **3** but switching to 7% methanol in dichloromethane as eluent gave **4** as an orange powder. Yield: 0.014 g (12%). ^1^H NMR (CDCl_3_) δ 7.33 (s, 4H, trz-*H*), 5.11 (s, 8H, Fc-C*H*_2_-trz), 4.14 (t, ^3^*J* = 1.7 Hz, 8H, Fc-*H*), 4.11 (t, ^3^*J* = 1.7 Hz, 8H, Fc-*H*), 2.74 (t, ^3^*J* = 6.6 Hz, 4H, trz-C*H*_2_-CH_2_), 2.23 (t, ^3^*J* = 6.6 Hz, 4H, C*H*_2_-C≡C), 1.76–1.86 (m, 8H, trz-CH_2_-C*H*_2_); ^13^C NMR (DMSO-*d*_6_) δ 145.8, 121.9, 83.6, 77.6, 69.3, 69.0, 65.7, 48.3, 27.7, 24.0, 17.9; HRMS–ESI (*m*/*z*): [M + Na]^+^ calcd for C_52_H_52_N_12_Fe_2_·Na, 979.3029; found, 979.3038.

### Bis(triazolium) macrocycle **5·2PF_6_**

The neutral macrocycle **3** (0.048 g, 0.10 mmol) was dissolved in dry dichloromethane (10 mL). Trimethyloxonium tetrafluoroborate (0.032 g, 0.22 mmol) was added and the orange solution stirred at room temperature under a nitrogen atmosphere overnight, during which time it took on a brown tinge. Methanol (1 mL) was added to quench the alkylating agent, and the mixture was evaporated to dryness under reduced pressure. The crude mixture was purified by preparative thin layer chromatography (silica, eluent: 17:2:1 CH_3_CN/H_2_O/sat. KNO_3(aq)_). The band containing the product was scraped from the plate and washed off the silica with the same solvent mixture, and the acetonitrile was removed under reduced pressure to give an orange aqueous solution. Addition of NH_4_PF_6_ (0.15 g in 0.5 mL H_2_O) caused the precipitation of a yellow solid. This was extracted into dichloromethane (2 × 15 mL), washed with water (2 × 15 mL) and thoroughly dried in vacuo to give **5** as a glassy orange solid. Yield: 0.0098 g (12%). ^1^H NMR (CD_3_CN) δ 8.32 (s, 2H, *H*_d_), 5.34 (s, 6H, *H*_c_), 4.37 (t, ^3^*J*_a,b_ = 1.9 Hz, 4H, *H*_b_), 4.24 (t, ^3^*J*_a,b_ = 1.9 Hz, 4H, *H*_a_), 4.19 (s, 6H, *H*_e_), 2.99 (t, ^3^*J*_f,g_ = 6.6 Hz, 4H, *H*_f_), 2.40 (t, ^3^*J*_g,h_ = 6.5 Hz, 4H, *H*_h_), 1.95–2.03 (m, partially obscured by residual acetonitrile solvent peak, 4H, *H*_g_). Compound lettering shown in Figure 2. ^13^C NMR (CD_3_CN) δ 145.1, 129.1, 81.6, 77.6, 71.2, 70.3, 66.6, 53.6, 38.7, 25.6, 22.7, 18.4; ^19^F NMR (CD_3_CN) δ −144.6 (heptet, *J*_P,F_ = 707 Hz); ^31^P NMR (CD_3_CN) δ −73.4 (d, *J*_P,F_ = 707 Hz); HRMS–ESI (*m*/*z*): [M + PF_6_]^+^ calcd for C_28_H_32_N_6_Fe·PF_6_, 653.1674; found, 653.1693

### Bis(triazolium)-bis(alkyne) **6·2BF_4_**

The neutral bis(alkyne) **2** (0.096 g, 0.20 mmol) was dissolved in dichloromethane (15 mL). Trimethyloxonium tetrafluoroborate (0.065 g, 0.44 mmol) was added and the orange solution stirred at room temperature under a nitrogen atmosphere overnight. Methanol (1 mL) was added to quench the alkylating agent, and the mixture was evaporated to dryness under reduced pressure. Purification by preparative thin-layer chromatography (silica, eluent: 10% methanol in dichloromethane) gave **6**·**2BF_4_** as an orange powder. Yield: 0.106 g (77%). ^1^H NMR (acetone-*d*_6_) δ 8.60 (s, 2H, trz^+^-*H*), 5.75 (s, 4H, Fc-C*H*_2_-trz^+^), 4.64 (t, ^3^*J* = 1.9 Hz, 4H, Fc-*H*), 4.42 (t, ^3^*J* = 1.9 Hz, 4H, Fc-*H*), 4.34 (s, 6H, trz^+^-C*H*_3_), 3.08 (t, ^3^*J* = 7.7 Hz, 4H, trz^+^-C*H*_2_-CH_2_), 2.41 (t, ^4^*J* = 2.6 Hz, 2H, C≡C*H*), 2.35 (dt, ^3^*J* = 6.8 Hz, ^4^*J* = 2.6 Hz, 4H, C*H*_2_-C≡CH), 1.93–2.02 (m, 4H, CH_2_-C*H*_2_-CH_2_-C≡CH); ^13^C NMR (acetone-*d*_6_) δ 145.0, 128.6, 83.5, 80.9, 71.6, 71.5, 71.2, 53.7, 38.1, 26.5, 22.8, 18.0; ^19^F NMR (acetone-*d*_6_) δ −150.9 (m); HRMS–ESI (*m*/*z*): [M + BF_4_]^+^ calcd for C_28_H_34_N_6_Fe·BF_4_, 597.2218; found, 597.2239.

### X-ray crystallography

Single crystal X-ray diffraction data for **3** were collected by using synchrotron radiation (λ = 0.6889 Å) at the Diamond Light Source, Beam I19. The diffractometer was equipped with a Cryostream N_2_ open-flow cooling device [19] and the data were collected at 100(2) K. A series of ω-scans was performed in such a way as to collect a half-sphere of data to a maximum resolution of 0.77 Å. Cell parameters and intensity data (including interframe scaling) were processed with CrysAlis Pro [20].

The structures were solved by charge-flipping methods with SUPERFLIP [21] and refined using full-matrix least-squares on F^2^ within the CRYSTALS suite [22]. All nonhydrogen atoms were refined with anisotropic displacement parameters. Hydrogen atoms were generally visible in the difference map, and their positions and displacement parameters were refined by using restraints prior to inclusion into the model employing riding constraints [23].

Crystallographic data for the structures have been deposited with the Cambridge Crystallographic Data Centre, CCDC: 859564 and 859565. Copies of these data can be obtained free of charge from The Cambridge Crystallographic Data Centre at http://www.ccdc.cam.ac.uk/data_request/cif.

### NMR titration protocols

Initial sample volumes were 0.50 mL and concentrations were 2.0 mmol L^−1^ of host. Solutions (100 mmol L^−1^) of anions as their tetrabutylammonium salts were added in aliquots, the samples thoroughly shaken and spectra recorded. Spectra were recorded at 0, 0.2, 0.4, 0.6, 0.8, 1.0, 1.2, 1.4, 1.6, 1.8, 2.0, 2.5, 3.0, 4.0, 5.0, 7.0 and 10 equivalents. Stability constants were obtained by analysis of the resulting data with the WinEQNMR2 [13] computer program, following the triazolium C–H protons in all cases.

### Electrochemistry protocols

Cyclic voltammetry was performed on an Autolab Potentiostat/Galvanostat model PG-STAT 12, controlled by General Purpose Electrochemical System Software v. 4.9 (Eco Chemie). A standard one-compartment three-electrode electrochemical cell, located inside a Faraday cage, was used with a glassy carbon solid-disc working electrode, a platinum-wire auxiliary electrode and an Innovative Instruments, Inc. LF-2 leak-free silver/silver chloride reference electrode. A 0.50 mM ferrocene sample was used in order to check the reference electrode and internal resistance of the equipment. The electrolyte solution used in all experiments was 0.10 M TBA·PF_6_ in CH_3_CN. CVs were recorded with a 1 s equilibration time and a step potential of 1 mV.

The receptor **3** was dissolved in 3.0 mL of the electrolyte solution, such that the receptor concentration was 0.50 mM, and cyclic voltammograms were recorded. Scan rates of 25, 50, 100, 200, 300, 400 and 500 mV s^−1^ were used in order to test for reversibility. Aliquots of TBA·Cl or TBA·BzO (as a 0.50 M solution of electrolyte solution) were added to the receptor solution, stirred and the cyclic voltammograms recorded at a scan rate of 100 mV s^−1^.

## Supporting Information

File 1 NMR-spectra of new compounds.

File 2 X-ray data of macrocycles **3** and **4**.

## References

[1] Rostovtsev V V, Green L G, Fokin V V, Sharpless K B (2002). Angew Chem, Int Ed.

[2] Tornøe C W, Christensen C, Meldal M (2002). J Org Chem.

[3] Juríček M, Kouwer P H J, Rowan A E (2011). Chem Commun.

[4] Li Y, Flood A H (2008). J Am Chem Soc.

[5] Li Y, Flood A H (2008). Angew Chem, Int Ed.

[6] Meudtner R M, Hecht S (2008). Angew Chem, Int Ed.

[7] Juwarker H, Lenhardt J M, Castillo J C, Zhao E, Krishnamurthy S, Jamiolkowski R M, Kim K-H, Craig S L (2009). J Org Chem.

[8] Mullen K M, Mercurio J, Serpell C J, Beer P D (2009). Angew Chem.

[9] Kumar A, Pandey P S (2008). Org Lett.

[10] Schulze B, Friebe C, Hager M D, Günther W, Kohn U, Jahn B O, Görls H, Schubert U S (2010). Org Lett.

[11] Cao Q-Y, Pradhan T, Kim S, Kim J S (2011). Org Lett.

[12] Casas-Solvas J M, Ortiz-Salmerón E, Giménez-Martínez J J, García-Fuentes L, Capitán-Vallvey L F, Santoyo-González F, Vargas-Berenguel A (2009). Chem–Eur J.

[13] Hynes M J (1993). J Chem Soc, Dalton Trans.

[14] Bard A J, Faulkner L R (1980). Electrochemical methods: fundamentals and applications.

[15] Evans N H, Serpell C J, Beer P D (2011). Chem Commun.

[16] Wong W W H, Curiel D, Lai S-W, Drew M G B, Beer P D (2005). Dalton Trans.

[17] Beer P D, Hayes E J (2003). Coord Chem Rev.

[18] Lee B-Y, Park S R, Jeon H B, Kim K S (2006). Tetrahedron Lett.

[19] Cosier J, Glazer A M (1986). J Appl Crystallogr.

[20] (2010). CrysAlis Pro.

[21] Palatinus L, Chapuis G (2007). J Appl Crystallogr.

[22] Betteridge P W, Carruthers J R, Cooper R I, Prout K, Watkin D J (2003). J Appl Crystallogr.

[23] Cooper R I, Thompson A L, Watkin D J (2010). J Appl Crystallogr.

