# Supplementary material for: A ferrocene redox-active triazolium macrocycle that binds and senses chloride
Source: Beilstein J Org Chem. 2012 Feb 13;8:246–52. doi: 10.3762/bjoc.8.25 (PMC3302085; doi:10.3762/bjoc.8.25)

Supporting Information

for

A ferrocene redox-active triazolium macrocycle that binds and senses chloride

Nicholas G. White and Paul D. Beer*

Address: Chemistry Research Laboratory, Mansfield Road, Oxford, OX1 3TA, United Kingdom

Email: Paul D. Beer - paul.beer@chem.ox.ac.uk

* Corresponding author

NMR spectra of new compounds

All spectra recorded at 293 K. The ^{1}H spectra at 300 MHz, ^{13}C at 75.5 MHz.

2

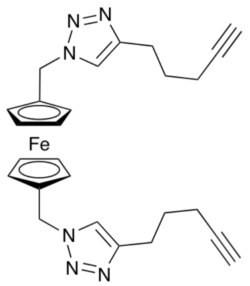

^{1}H ($CDCl_3$)

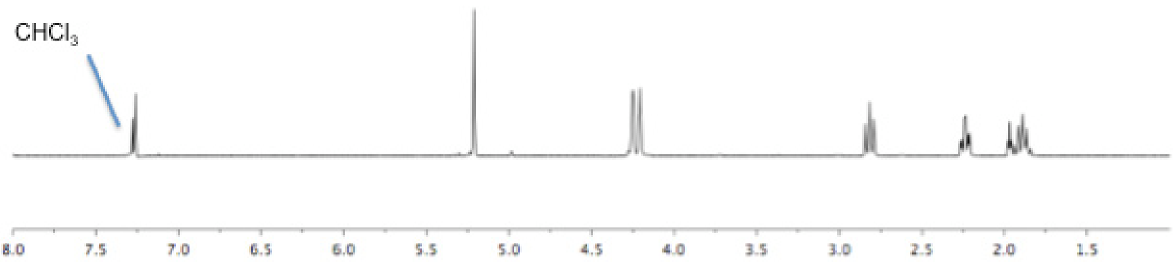

^{13}C ($CDCl_3$)

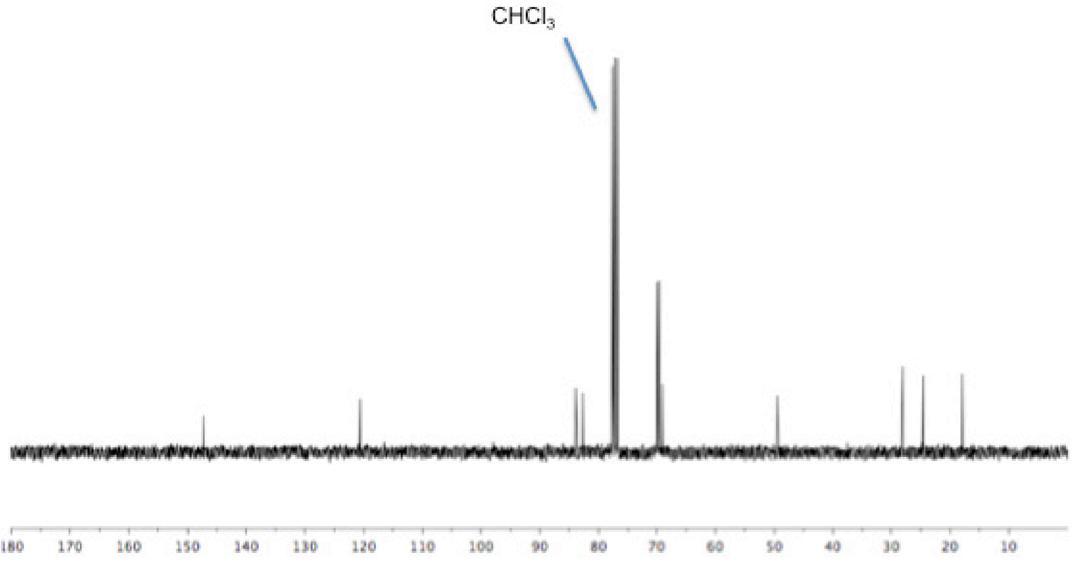

3

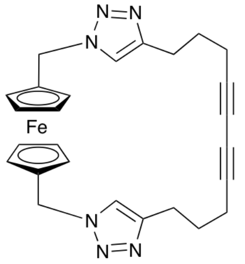

^{1}H $(CDCl_3)$

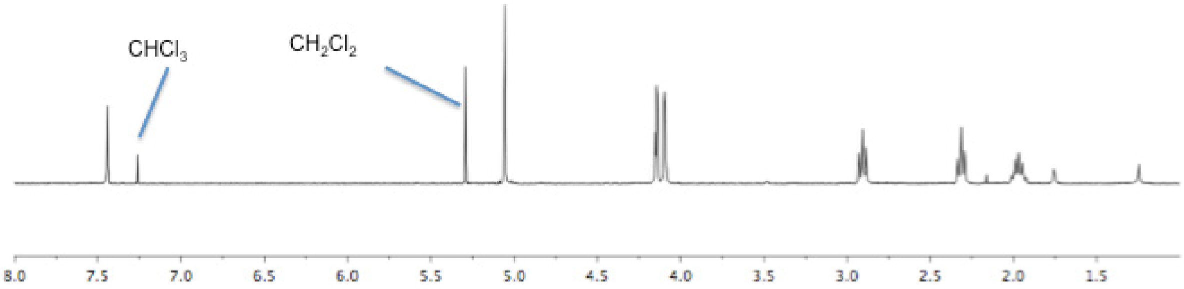

^{13}C $(CDCl_3)$

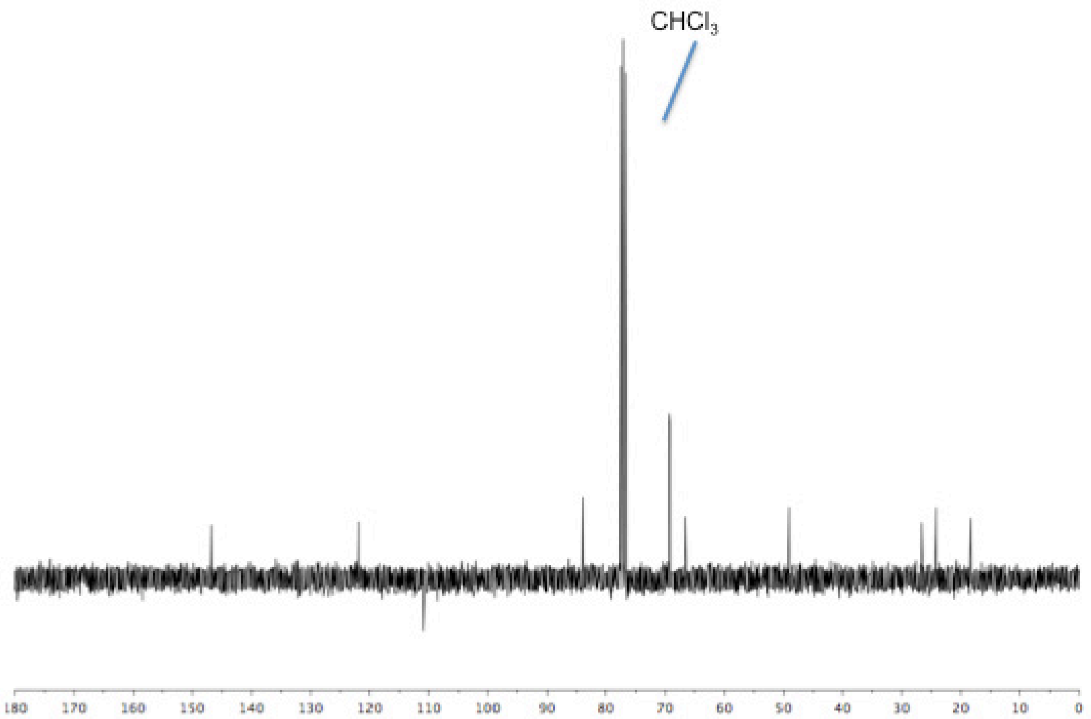

4

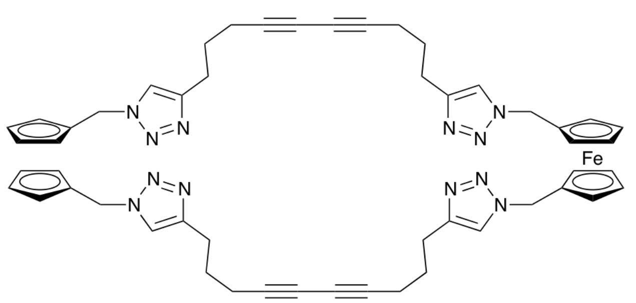

^{1}H (acetone-d_6)

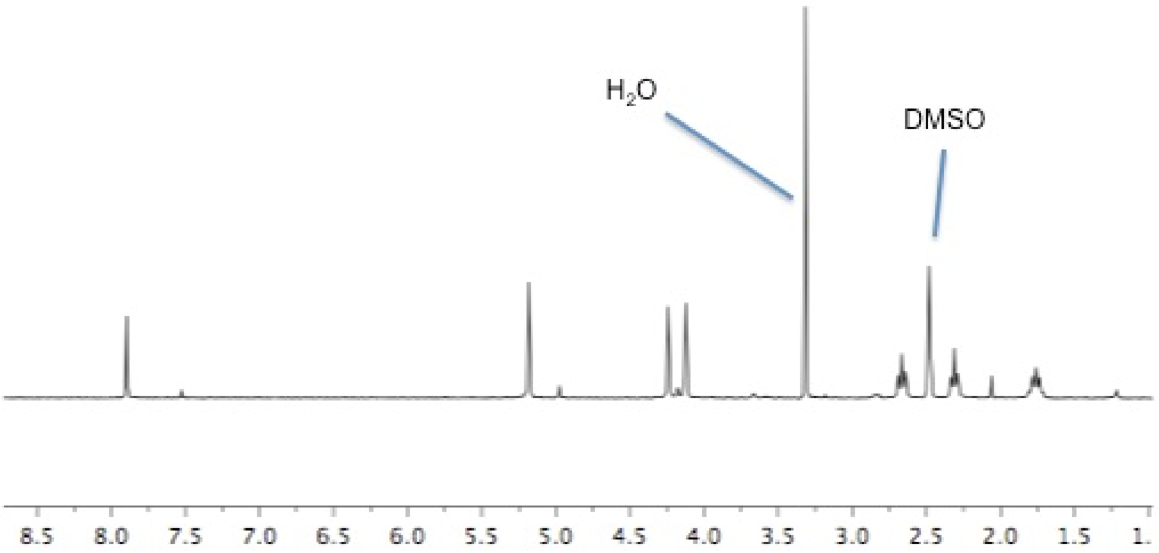

^{13}C (acetone-d_6)

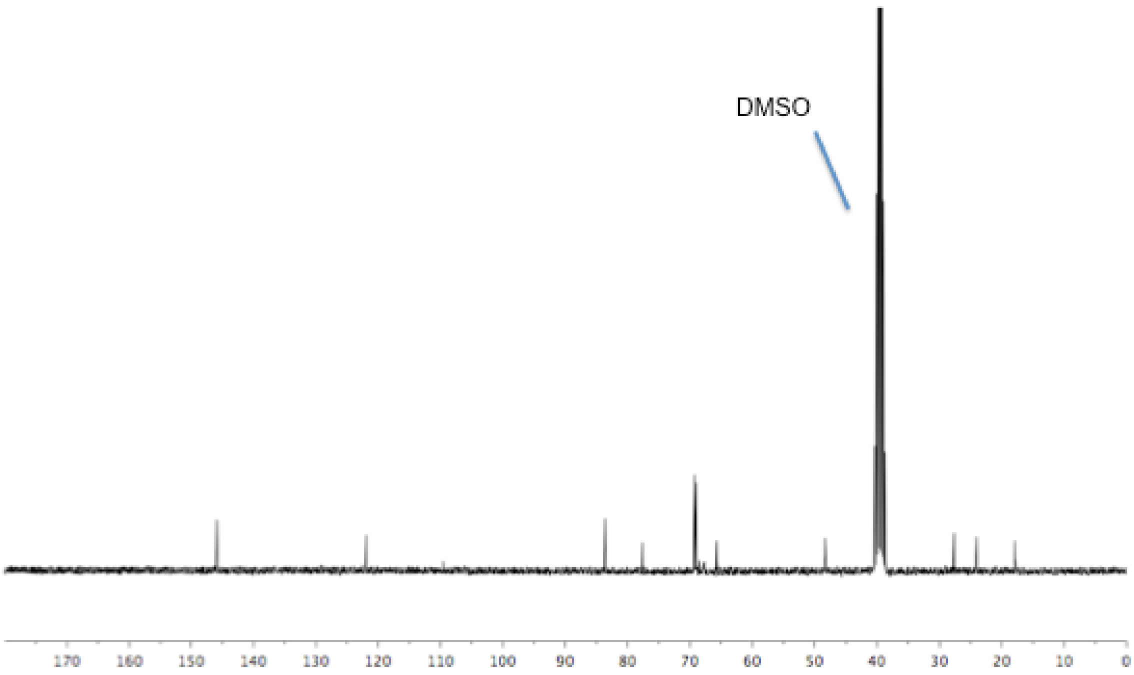

5

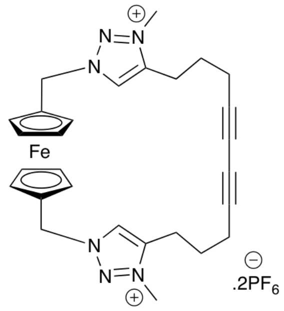

N
N
N
Fe
N
N
N
.2PF6

^1H (CD_3CN)

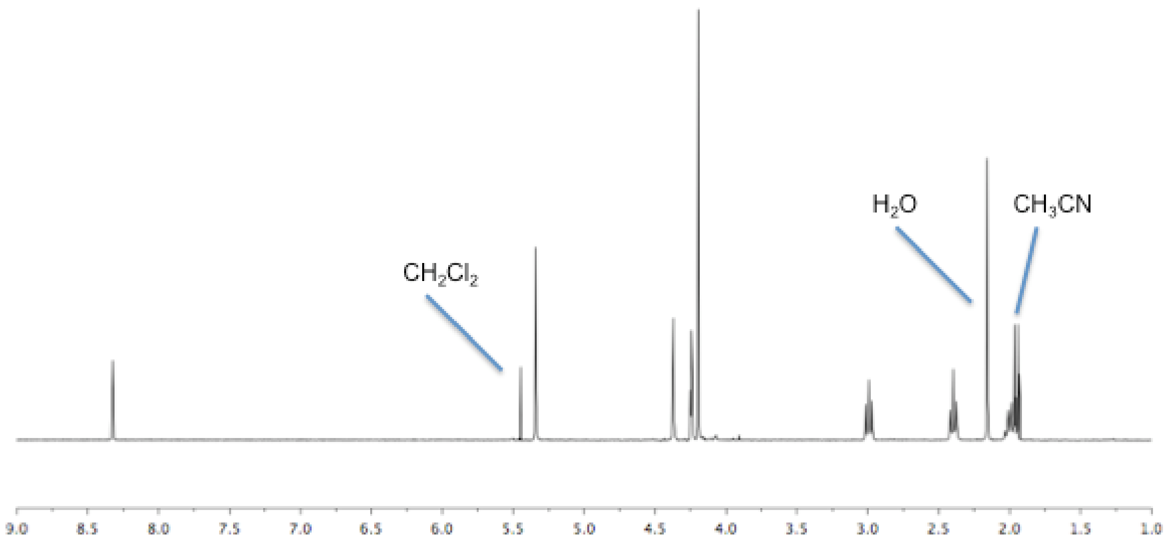

CH2Cl2
H2O
CH3CN
9.0
8.5
8.0
7.5
7.0
6.5
6.0
5.5
5.0
4.5
4.0
3.5
3.0
2.5
2.0
1.5
1.0

^{13}C (CD_3CN)

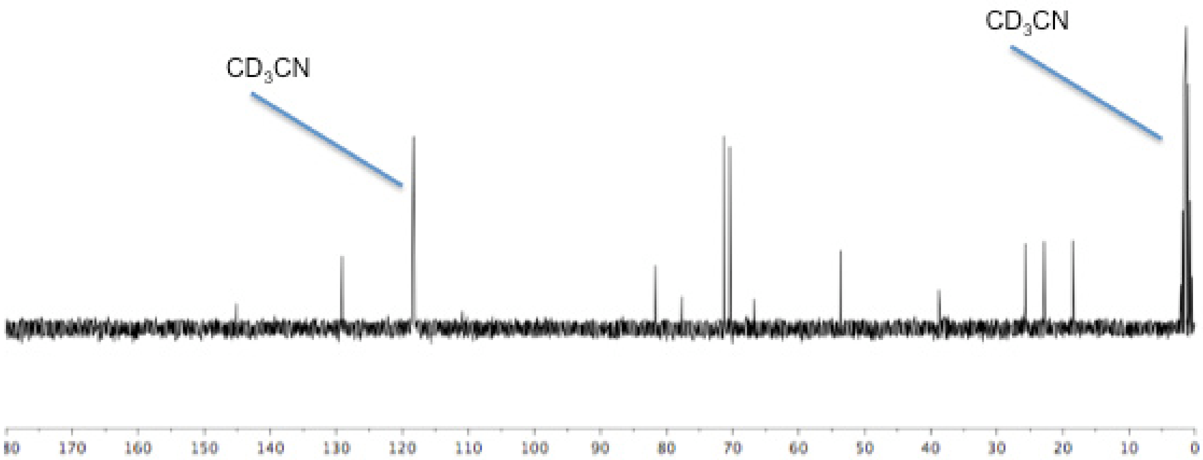

^{19}F (CD_3CN)

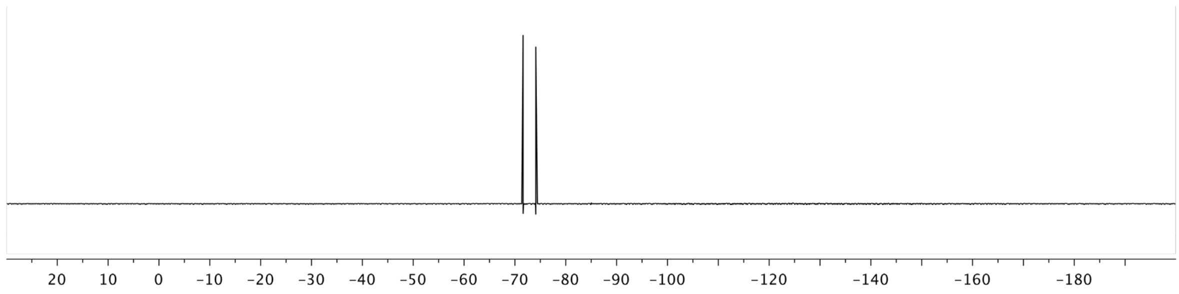

^{31}P (CD_3CN)

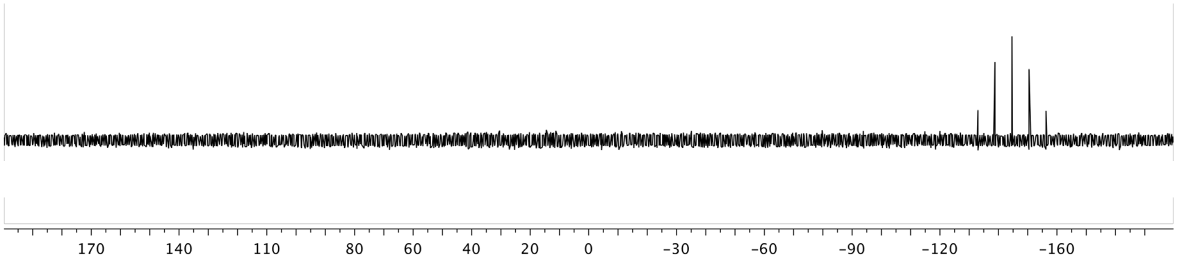

6

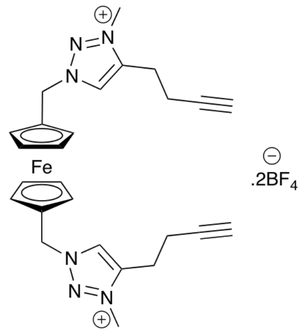

^{1}H (acetone-d_6)

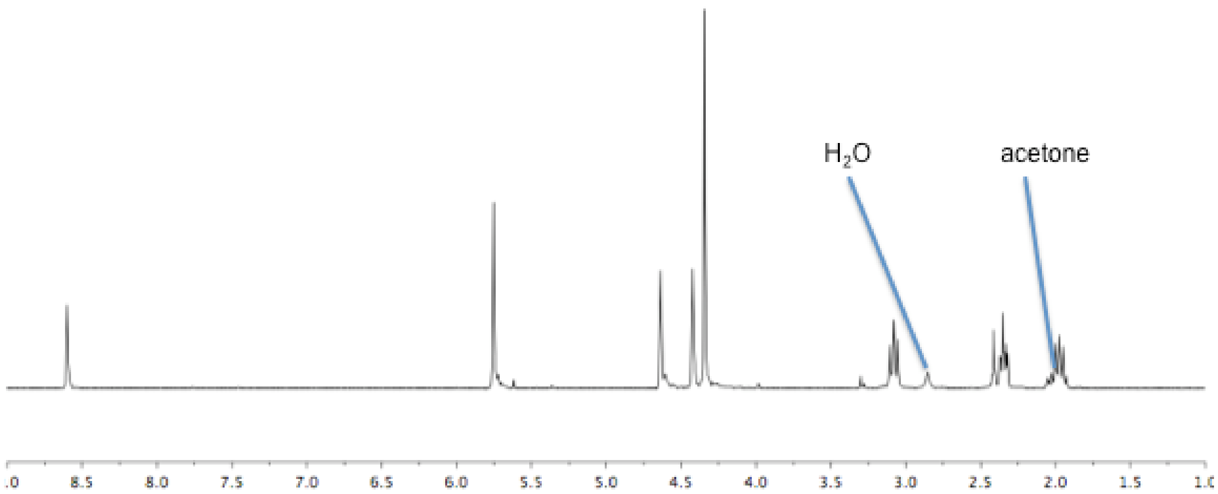

^{13}C (acetone-d_6)

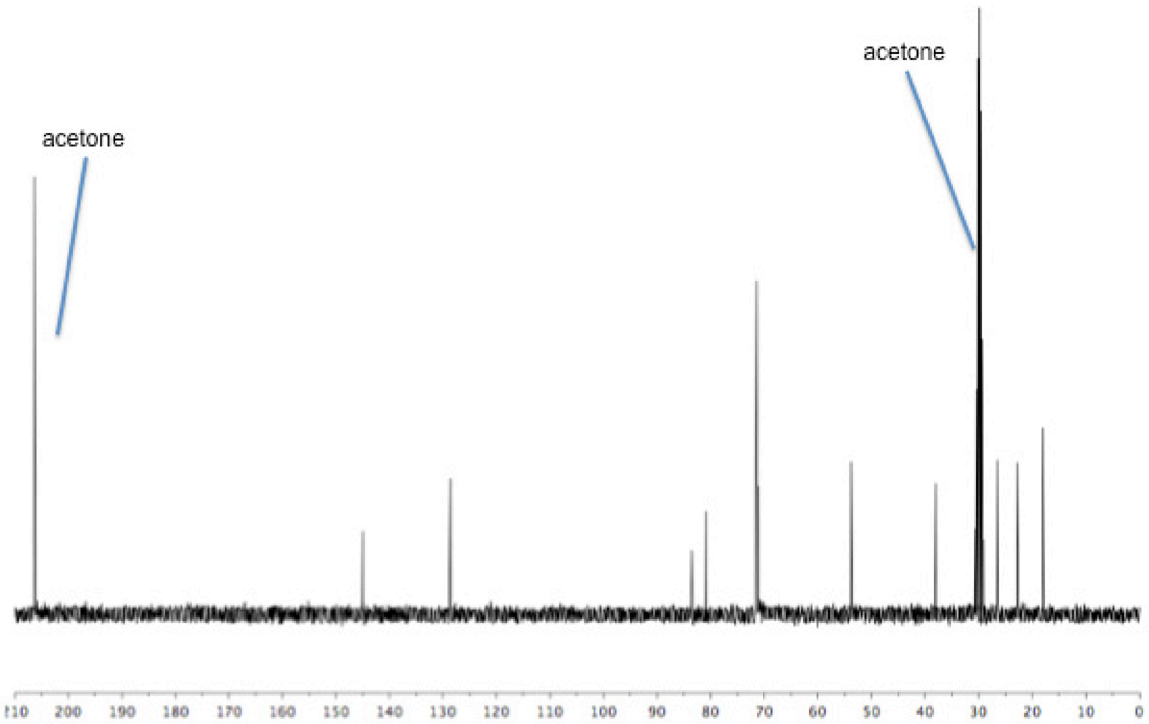

^{19}F (acetone-d_6)

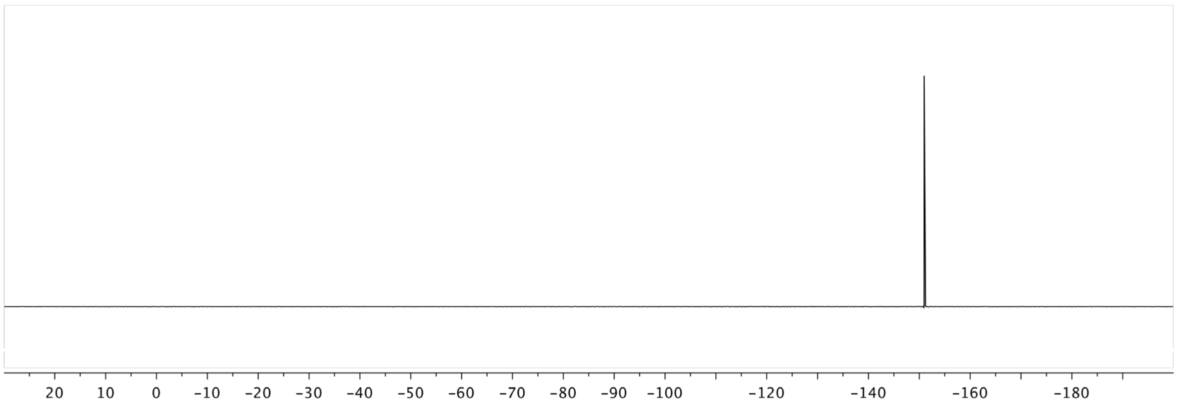

Supplement: File 1 — NMR-spectra of new compounds. [file Beilstein_J_Org_Chem-08-246-s001.pdf]
